# Seabird parents provision their chick in a coordinated manner

**DOI:** 10.1371/journal.pone.0189969

**Published:** 2018-01-10

**Authors:** Katarzyna Wojczulanis-Jakubas, Marcelo Araya-Salas, Dariusz Jakubas

**Affiliations:** 1 University of Gdańsk, Faculty of Biology, Department of Vertebrate Ecology and Zoology, Gdańsk, Poland; 2 New Mexico State University, Las Cruses, New Mexico, United States of America; 3 Laboratory of Ornithology, Cornell University, Ithaca, New York, United States of America; Norsk Polarinstitutt, NORWAY

## Abstract

Pair collaborative behavior may play an important role in avian reproduction. However, evidence for this mainly comes from certain ecological groups (e.g. passerines). We studied the coordination of parents in foraging and its effect on food provisioning rate and chick growth in a small seabird, the Dovekie (Little auk, *Alle alle*). The species exhibits a dual foraging strategy, where provisioning adults make foraging trips of short (mean ~2 h; to provide food for the chick) and long duration (mean ~ 13 h; mainly for adults self-maintenance, although the food is also brought to the chick). We expected that offspring would benefit if parents coordinate their foraging patterns: one making short trips in the time when the other performing the long one. We examined this hypothesis using Monte Carlo randomization tests on field data collected during observations of individually marked birds. We found that parents did indeed adjust provisioning, making their long and short trips in an alternating pattern with respect to each other. Furthermore, we found that a higher level of coordination is associated with a lower variability in the duration of inter-feeding intervals, although this does not affect chick growth. Nevertheless, our results provide compelling evidence on the coordinated behavior of breeding partners.

## Introduction

Bi-parental care in birds has long been viewed as a tug-of-war between pair members [1]. This is because the costs of parental care in terms of reduced parent survival or fecundity are assumed to be high [2, 3], making the parent prone to shifting the burden of offspring care onto the mate [1, 4]. How the conflict between mates over parental care is resolved has been the subject of numerous studies. The classical model [5], examining the issue over evolutionary timescale, has demonstrated that bi-parental care is an evolutionary stable strategy if each parent, working independently, provides a fixed amount of parental care (“sealed bid”), given the effort of its mate. Other models examining the issue on a behavioural timescale considered the possibility that parents can adjust their effort in response to that of their partners’ [6–10]. All these models predict that the conflict leads parents to reduce their investment in care, with negative consequences for their offspring. The most recent model [11] has demonstrated that a simple form of conditional cooperation may ameliorate the conflict between parents over offspring care, with the model’s prediction receiving empirical support.

The key point of the concept of sexual conflict over parental care is that mates share the benefits but not the costs of their partner’s effort [8, 10]. When both mates might be adversely affected by over-investment by the partner, the conflict, if still present, is not that apparent. This is the case in many socially monogamous species, where parents stay together for multiple breeding seasons, sharing both benefits and costs of their partner’s workload [12]. For such groups, the parents relation may be considered as a ‘family firm’, where both partners—the firm owners—work together for multiple seasons to maximize a common product (the offspring) [13]. As such, parents may achieve a more efficient breeding outcome by coordinating their reproductive effort.

Partner effort coordination has been assumed to contribute in increasing reproductive success in species demonstrating long-term pair bonds, such as seabirds and waterbirds, following the hypothesis known as “mate familiarity effect” [14]. The hypothesis has largely been tested indirectly, only documenting the improvement in reproductive success over the duration of a pair bond, rather than by empirically measuring behavioral coordination of parental duties [15]. Nevertheless, a growing number of studies on passerines show that parents coordinating their parental performance enhance their reproductive outcome (e.g. [12, 15, 16, 17]), indicating a beneficial role of pair coordination [13].

Pelagic seabirds exhibiting a dual foraging strategy (e.g. some species of albatrosses, petrels, penguins, auks [18–20]) offer an interesting viewpoint in considering coordinated efforts of breeding partners. A dual foraging strategy is composed of long foraging trips, primarily serving adult self-maintenance (some food is also brought to the chick), alternated with short trips that serve solely to provision the offspring. From the offspring’s perspective, the adults’ long trips represent extended periods of waiting for food whereas the short ones represent a great amount of food over a short period of time. Both situations may be disadvantageous for the offspring. During long trips, the risk of offspring starvation increases considerably, whereas during short trips the young birds may face a difficulty assimilating large amounts of food (e.g. [21, 22]). This may be particularly profound when both parents make their long and short trips simultaneously. This may then affect the chick’s body condition, leading to starvation (if the fasting period is too long) or obesity [21, 22]. Chick obesity is a strategy employed by many albatross and petrel species to survive the long fasting periods, as even short trips in these species last a day and long trips may last a few days [21, 22]. This strategy is not free of costs, however, leading to a prolonged chick growth rate [22]. When parents offset their foraging trips so that one makes a long trip while the other makes short ones, the offspring may assimilate food more easily and grow and develop faster. Accordingly, chick growth and development, and ultimately breeding success, may be related to the level of trip coordination between the parents. In such a context, one may expect a coordinated performance of pair members by avoiding an overlap of long trips. Indeed, such a coordinated manner of food provisioning has been reported in the Wedge-tailed shearwater *Puffinus pacificus* [23]. However, neither coordination or it’s impact on offspring condition were the focus of the study.

In this study, we examined the foraging pattern of Dovekie parents (Little auk, *Alle alle*). This is a small long-lived seabird, with long-term pair bonds, and long and extensive bi-parental care [24]. The parents equally share their incubation duty for four weeks [25] and both brood and feed the chick at a similar rate for 3–4 weeks [26]. Only at the very end of the nesting period (1–3 days before fledging) the female deserts the colony, while the male continues to provide the food and accompanies the fledgling during its colony departure [27, 28]. The feeding frequency of Dovekie is high (on average 3.5 feedings per parent per 24 h; e.g. [29]), which is probably related to the high metabolic rate of the species [30]. Regardless of the environmental conditions, Dovekie consistently exhibits a bimodal foraging pattern during the chick rearing period (e.g. [31]), switching from unimodal to this bimodal pattern immediately after the chick has hatched [32]. The proportion of time spent by birds on short and long trips is stable throughout the chick rearing period [33], although the alternating pattern of long and short trips varies (i.e. number of short and long trips performed in a row) spatially and temporarily [31–33]. It has been shown that the body mass of adults increases during the long trip and decreases during the short trips [34], indicating that this bimodal foraging strategy is crucial for adult self-maintenance.

We hypothesized that an optimal strategy could be achieved through the coordination of long and short foraging trips between males and females, with one making a long trip while the other makes short ones. We examined this hypothesis by investigating the pattern of male and female provisioning using a randomization procedure to compare it with the pattern expected by chance. We also expected that coordination of provisioning should reduce the time-intervals between feedings, which in turn, should positively affect chick body condition.

## Materials and methods

We conducted the study during the chick rearing period (July–August) in two consecutive years (2009 and 2010) in Magdalenefjorden, NW Spitsbergen (79^o^35’N, 11^o^05’ E), one of the biggest Dovekie breeding aggregations in the Svalbard archipelago (ca 18 000 breeding pairs, [35]). The nearby shelf sea area, though supplied with warm Atlantic water from the West Spitsbergen Current, still remains under the influence of Arctic waters from the South Cape Current [36]. As such, the shelf area constitutes a good foraging ground for local birds [36]. The birds also utilize the marginal ice zone 90–150 km away, which seems to be more profitable than the area in the close vicinity of the colony (e.g. [37]).

To establish the chick provisioning pattern of the studied Dovekies, we observed uniquely marked individuals over 48 h non-stop observations. We performed two sessions of observation in 2009 and three in 2010. To mark the birds, we captured them on nests during the late incubation period, color-ringing and painting individually recognizable signs on the birds’ breast feathers with permanent, waterproof markers (Sharpie, USA). This double marking system allowed us to easily and reliably identify the birds while they were present in the colony. We took a small blood sample (ca 20 μL) from the brachial vein of all birds handled and preserved it in 1 mL of 96% ethanol for later DNA-based sex identification, as there is no apparent sexual difference in size or plumage allowing the sexes to be reliably distinguished [38]. We released the bird unharmed directly into the nest after ca 10 min of handling. In total, we marked and further observed both members of 18 breeding pairs in 2009 and 22 pairs in 2010. Fifteen of those pairs were the same in both seasons.

The two observation sessions in 2009 took place when the chicks from the focal nests were on average 9 (range: 5–18) and 17 days old (10–26), respectively. The three observation sessions in 2010 were performed when the chicks were on average 11 (range: 6–16), 20 (15–25) and 24 (20–29) days old, respectively. During each 48 h observation session, we continuously observed the colony area with the focal nests. Two persons sitting behind a blind ca 20 m from the colony edge (a distance allowing the birds to be observed without their behavior being affected) performed the observations. The observers used binoculars (10x35) to confirm the birds’ identity. Birds were observed continuously and their presence/absence was noted every 10 min. This is because, although the birds stay at the colony between foraging trips for a longer period of time (on average 49 min; min-max: 10–80 min), the exact moment of their colony departure is sometimes difficult to observe. Thus, the established 10 min “time-window” enabled us to monitor the birds’ presence and estimate the moment they departed from the colony for their foraging trip within 10 min accuracy. We considered a foraging trip to be the period of the bird’s absence, after which it reappeared in the colony with a full gular pouch (i.e. indicating foraging). The birds returning from foraging trips usually entered the nest immediately (average latency = 7 min). Birds were marked from nests situated in close proximity to each other within a small (200–300 m^2^) colony patch, allowing the two observers to track all the marked birds. Strong wind and heavy rain can affect the feeding frequency of Dovekie [39], but in the present study the weather conditions were mild (little wind, little or no rain). Conditions were similar both the day before and during all the observations in both seasons, so we did not consider weather as a factor in our analyses.

To establish the chick hatching date for marked pairs, their nests were monitored every 2 days, starting from ca 3 days before the expected onset of hatching in the colony. To control chick body condition and survival, we monitored the target nests and weighed the chicks every 3 days, beginning from the 13^th^ or 14^th^ day of life until fledging, using an OHAUS (Parsippany, New Jersey, USA) electronic balance (accurate to 0.1 g). We considered a nestling to have fledged when it disappeared from the nest after the 21^st^ day of life (on average age of the chick departure is 26^th^ day of life, range: 21–31; [26]). We monitored the nests and weighed the chicks between the observation sessions, avoiding potential disturbance that could affect feeding patterns.

We performed molecular sexing based on DNA extracted from the blood samples. To extract DNA we used the Blood Mini kit (A&A Biotechnology, Gdynia, Poland), following evaporation of the alcohol. We used the primer pair 2550F and 2718R according to the protocol described by [40], applying a 50°C annealing temperature in the polymerase chain reaction. The primers amplify introns on the CHD-W and CDH-Z genes located on the W and Z avian sex chromosomes [41]. The difference between the two fragments (ca 200 bp) was clearly visible in UV light when separated on 2% agarose gel and stained in ethidium bromide.

### Data analysis

**Fig 1:**
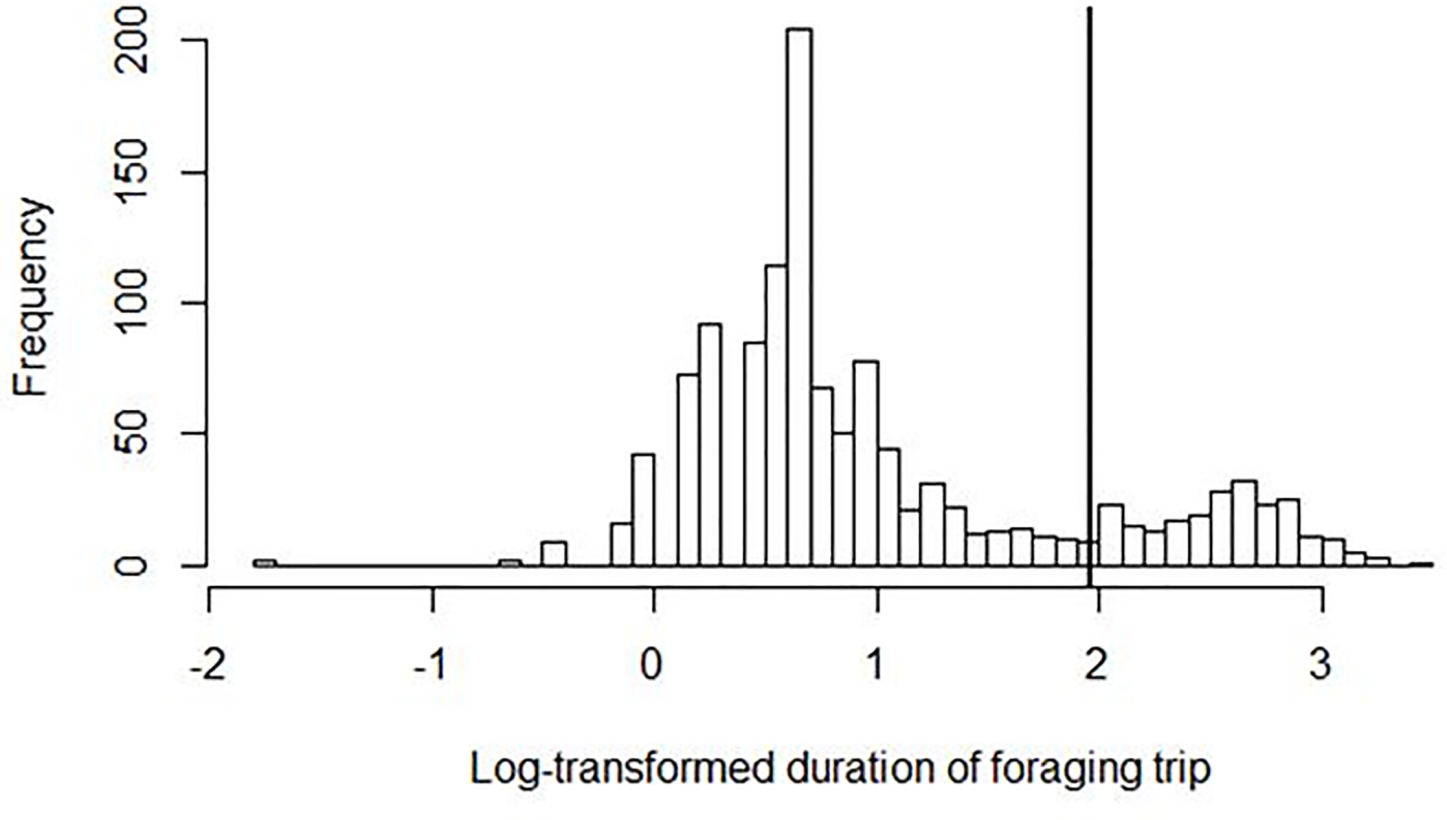
Distribution of log-transformed duration of the dovekie foraging trips. The vertical line indicates a cut-off value (log of 7.1 hours) to separate short and long trips, following the algorithm that minimizes the sum of the variances of both types of trips given their log-normal distribution [31].

We calculated the duration of foraging trips based on the 10 min “time-windows” for which the presence/absence status of the marked individuals in the colony had been established. To determine a cut-off value for separating short (hereafter ST) and long trips (LT), we applied the method used previously by [31], where the best cut-off value is that which minimizes the sum of variances of both trip types, given their log-normal distribution. In our study, the derived cut-off point was 7.1 h (Fig 1). One of three categories (ST, LT or presence at the colony (CO)) were assigned to every 10 min “time-window” for each individual. In our fixed 48 h observation schedule it is axiomatic that some of the trips start or end beyond the observation period, which could potentially bias further analysis. To address this methodological constraint we excluded records of trips that started before the onset of the observation, or ended after it was terminated, along with the respective records for the partner. Nevertheless, given the mean duration of ST (1.9 h) and LT (12.8 h), we were able to keep records of individuals for at least one completed LT and few completed ST per bird (S1 Data and S2 Data).

Pair observation (the whole 48 h observation session for a given pair) where a color mark in at least one of the pair members had faded (posing possibility of overlooking the bird) were excluded from the analysis. We also excluded observations of pairs in which the female was in the process of brood desertion or had already abandoned the brood; i.e. the female performed a single feed during the whole 48 h period or did not appear at all. This was because the process of female desertion, although interesting *per se*, could impair analyses designed to test the pattern of provisioning by the two parents. In total we excluded 23 pair-observations. Therefore, the number of 48 h sessions varied slightly among pairs (median = 3, range 1–5 sessions per pair, (see S1 Table for details)).

To examine how partners match their foraging trips we analyzed the frequency of 10-min “time-windows” in which one pair member was on ST while the other was on LT. To test the statistical significance of this frequency, we used a Monte Carlo randomization approach (e.g. randomization that does not necessarily generate all possible combinations, [42]; the protocol in S1 R-script). Randomization methods provide a robust alternative to analyze data that do not conform to a conventional statistical approach ([43, 44]). For the randomization, we built a routine to shuffle the string of the three activity categories (ST, LT and CO), with each string (COi, STj, LTk) being of various durations. As each foraging trip ends at the colony the routine kept one randomly sampled CO string between foraging trip strings (irrespective of their nature–ST or LT). Since the pattern of LT and ST performed in a row varies temporally within individuals as shown in [31–33], we assumed that in the given period of time (48 h) the strings of ST and LT are independent. Consistently, we did not constrain the number of ST and LT performed in a row. All strings were sampled without replacement resulting in new overall series. These randomized series were then compared to the observed data using the statistic of the number of 10 min “time-windows” when one pair member is on ST while the other is on LT. We calculated the p-values as the proportion of expected random values that were higher than the observed value for each observation period. Both male and female strings were shuffled 10 000 times, and the randomization was performed based on the individual- and observation-specific pool of trip types and duration. We ran separate tests for each nest/observation session. To produce a single p-value for breeding pairs with several observation periods we combined the probabilities using the Z-method [45], although the alternative Fisher's and logit methods produced qualitatively equivalent results (see code in S1 R-script). This was performed to generate an overall p-value and avoid the potential problem of pseudoreplication.

We also applied a Monte Carlo randomization test to assess the probability of an even distribution of the chick feedings during the observation period. For that we calculated the coefficient of variation of the duration of inter-feeding intervals, with feedings determined to occur at the end of each foraging trip (confirmed by observations of individuals entering the nests with full gular pouch, after going back from the foraging trip). We compared this coefficient with the distribution of the coefficient of variation expected by chance (i.e., created in the randomization procedure) to obtain a p-value. As previously, we ran separate tests for each nest/observation session and combined the probabilities to produce a single p-value for breeding pairs with several observation sessions, avoiding pseudoreplication.

To examine the relationship between parent coordination and chick age, as well as the effects of coordination on the variation in time-intervals between the feedings and chick body condition, we first calculated an index of coordination, hereafter “coordination level”. The coordination level represents the proportional difference between the observed (obs) and expected (exp) number of 10 min “time-windows” in which one pair member was on ST while the other was on LT according to respective randomization procedure ([obs–exp] x exp^-1^). We calculated the coordination level for each nest/observation separately, also calculating the variation in inter-feeding interval duration relative to chance using the same method.

All chicks survived until fledgling so we only examined the chick growth parameters: raw chick body mass on day 14–16 of life (when the chick begins to exercise its wings outside the nest chamber. Until this age the chick remains in the nest, expending energy mainly on growth and thermoregulation [24]), peak body mass (the highest mass noted per chick), fledging body mass (the last mass measured before the chick’s departure from the colony), mass recession (difference between peak and fledging mass; after [24]), the day of life when the chick achieved its peak body mass, and the day it fledged. All these variables have been found to be effective growth indicators (e.g. [46]), even more efficient than the widely used growth-curve analysis [47]. We also calculated two values of the Specific Growth Rate using the formula SGR = (ln (m_2_)—ln (m_1_)) x ((t_2_ –t_1_) x 100)^-1^, where m_1_ and m_2_ are the body masses at times t_1_ and t_2_ respectively, and t_1_ < t_2_ [48]_._ We used chick body mass on day 14–16 of life (m_1_) and peak body mass (m_2_) to calculate SGR_1_, and peak body mass (m_1_) and fledging body mass (m_2_) to calculate SGR_2_. Since all parameters were correlated to each other, we reduced dimensions performing Principal Component Analysis on all Z-transformed (scaled) body condition variables. We found that the first two principal components (PCs) together explained 62% of the variation in chick body condition, and were further used in the analysis.

In four separate analyses we examined: a) the relationship between coordination level and chick age, b) the relationship between coordination level and the variation in duration of actual inter-feeding intervals relative to chance, c) the effect of pair level coordination on chick body condition and d) the effect of variation in the duration of inter-feeding intervals on chick body condition. In all these analyses we used linear mixed models fitted with maximum likelihood, including breeding pair as a random effect (random intercept). Since the number of feedings affects the chick’s growth, we also included total number of feedings per 48 hours in the model, testing the effect of coordination on the chick body condition; the number of feedings were not correlated with the response variable (Pearson correlation, r = 0.03; t = 0.14, df = 28, P = 0.89).

All the analyses were carried out in R 3.2.0 [49]. Linear mixed models were done using the R package *lme4* [50]. Combined probabilities were obtained using the package *metap* [51]. Statistical significance was set at α = 0.05.

### Ethical note

All the fieldwork was performed by ourselves (KWJ and DJ; both of us have the relevant qualifications and experience). We used non-toxic markers to mark the birds and applied sterile, disposable equipment for taking the blood samples. We released all birds unharmed after ca 10 min of handling. We observed and handled the birds under permission of the Norwegian Animal Research Committee and the Governor of Svalbard (2007/00150-9, 2007/00150-11).

## Results

**Fig 2:**
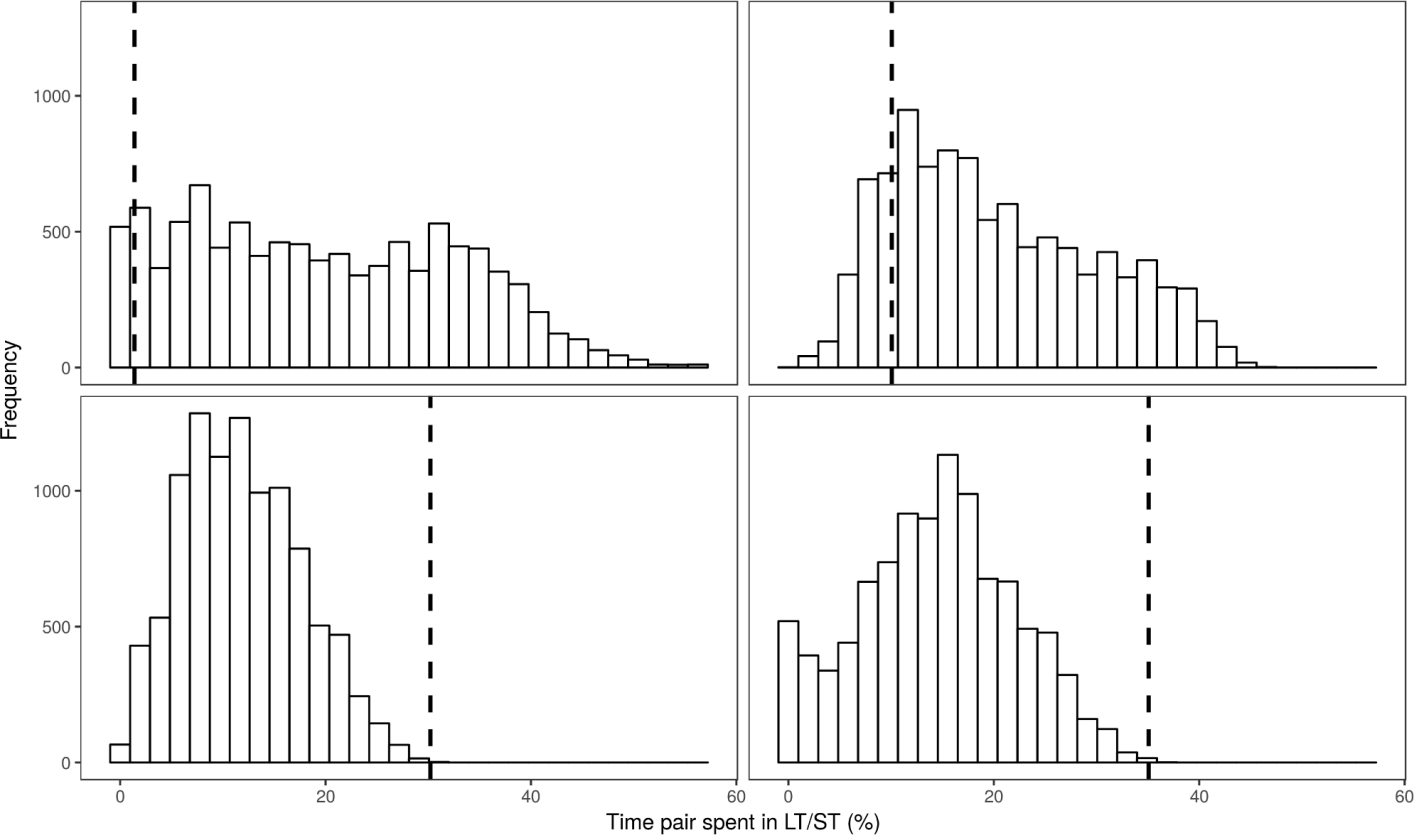
Four examples (pairs/observation sessions) of distribution of the random proportions of 10 min time-windows (during the 48 h observation periods) in which one pair member was on short trip (ST) while the other was on long trip (LT) (generated in the randomization procedure). Dotted line denotes the observed value.

We found a relatively high proportion of 10 min “time-windows” (during the 48 h observation periods) in which one pair member was on ST while the other was on LT (on average ± SD: 23.7% ± 14.1%, N = 25 pairs, 79 periods; Fig 2). This proportion was more frequent than expected by chance according to our randomization procedure (Z = 4.3, P < 0.0001), indicating coordinated provisioning by pair members.

The duration of inter-feeding intervals within observation periods lasted on average ± SD: 175 ± 45.5 min (N = 25 pairs, 79 periods). Variation of these inter-feeding intervals was significantly lower than expected by chance according to our randomization procedure (Z = 2.6, P = 0.004), which indicates an even distribution of feedings over time.

**Fig 3:**
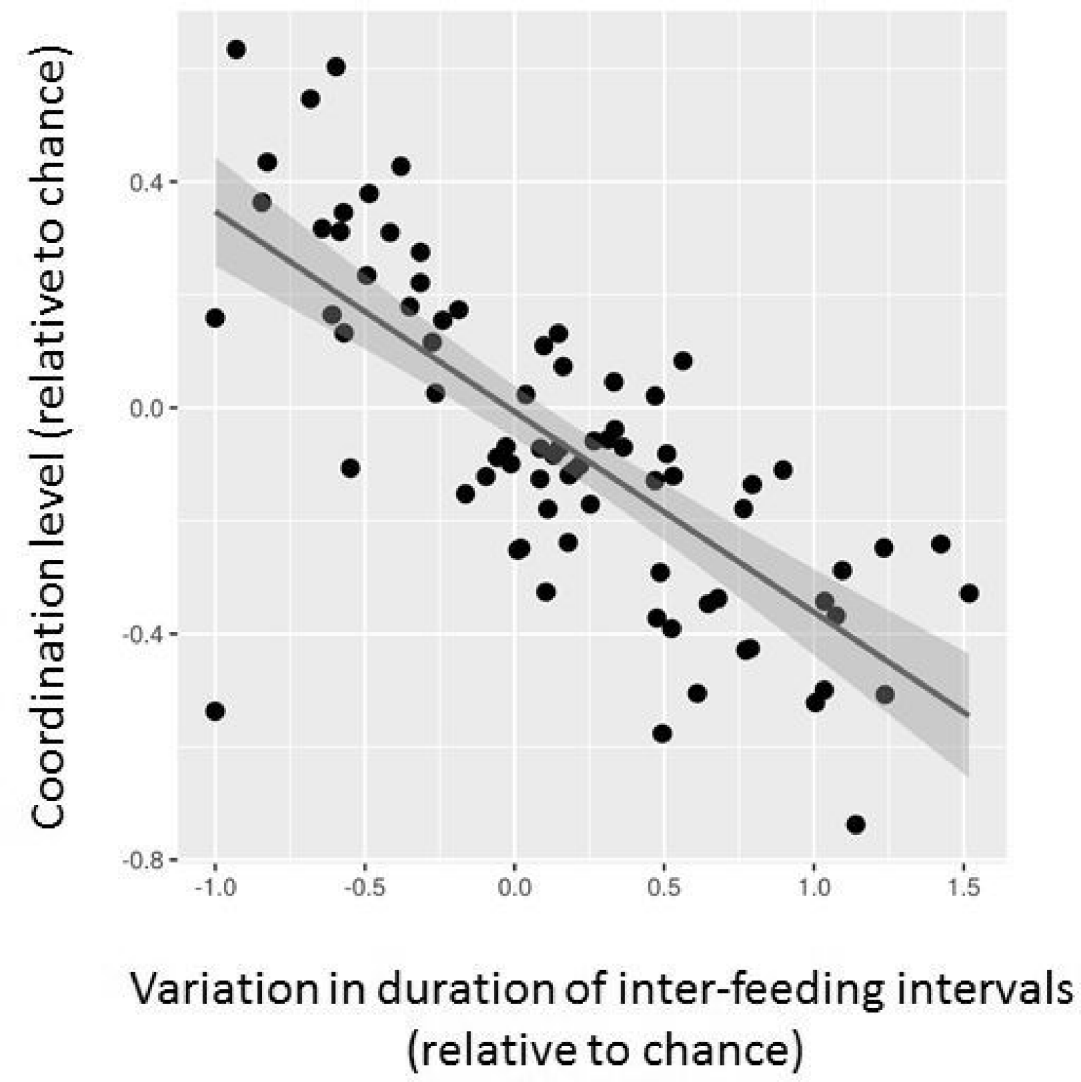
Relationship between the coordination level of provisioning pair members and variation in duration of inter-feeding intervals. Both variables are expressed relative to the value expected by chance according to the randomization test.

We did not find a significant relation between the coordination level of the two parents and chick age (F_1,53_ = 0.01, P = 0.91). However, the coordination level was significantly associated with variation in inter-feeding interval duration; the variation decreasing with an increasing coordination level (i.e. trend to more equal distribution of feedings through time; F_1,24_ = 11.93, P < 0.0001; Fig 3).

No significant effect was found between parental coordination and chick growth. None of the principal components were associated with coordination level (PC1: F_1,19_ = 0.13, P = 0.72; PC2: F_1,19_ = 1.14, P = 0.32) or variation in duration of inter-feeding intervals (PC1: F_1,19_ = 0.34, P = 0.57; PC2: F_1,19_ = 0.00003, P = 0.99), after controlling for pair identity and number of feedings.

## Discussion

Our results clearly showed that Dovekie parents adjusted the timing of ST and LT in regard to those of the partner, avoiding performing LTs at the same time and thereby securing a regular food delivery to their chick. Owing to the coordination, the chick was fed more steadily through time, although it did not affect its growth parameters.

So far, apart from the present study, a coordinated manner of food provisioning has been reported in only a single seabird species, the Wedge-tailed shearwater. Breeding shearwaters also exhibit a dual foraging strategy, avoiding performing their LTs at the same time as their partner [23]. However, while the Wedge-tailed shearwaters avoided overlapping foraging trips lasting a few days (~8 d), Dovekie parents exhibited a similar pattern within a shorter time frame (mean ~ 13 h, present study). This indicates that, regardless of the duration of the foraging trips, the parents tend to coordinate their provisioning with each other.

A number of studies investigating coordination in non-seabirds, such as passerines [11, 12, 15, 16, 17, 52] and parrots [53], have also reported that breeding parents exhibit a pattern of feedings in relation to each other. In some of these cases, males and females arrive/enter the nest at roughly the same time [12, 16, 17, 52, 53]. Such synchronicity is expected to reduce predation pressure (by reducing overall number of nest visits, where each may reveal the nest location to a predator, [16, 54]). It has also been suggested that the synchronization of parent visits allow them to better distribute the food among the offspring [12,55]. In other cases, male and female alternate their feeding in relation to each other, with each parent seeming to speed up its consecutive feeding visit if the partner had recently visited the chicks, but slowing down if only she/he visited in turn [11, 15]. Such a pattern indicates a form of reciprocity, which seems to be a key mechanism in ameliorating sexual conflict over parental care and allowing the parents to cooperate [15]. All these patterns of parental coordination reported in non-seabird species are different from that observed in the Dovekie, where the parents alternate not a single feeding but the whole series of feedings performed on their own. The interspecies variation highlights the need to examine the parental performance across a wide range of ecological groups to fully understand parental investment in birds.

We expected that the level of coordination of parental provisioning would affect duration of inter-feeding intervals, and would consequently affect chick growth (e.g. [22]). Indeed, we found that a lower level of coordination increased variation in duration of inter-feeding intervals. This suggests a potential mechanism underlying pair coordination, i.e. parents that coordinate provisioning reduce duration of periods when chicks are waiting for food. The relationship between the level of pair coordination and duration of inter-feeding intervals could be inherently linked. However, regardless of the direct mechanism, the outcome of coordination is more regular and frequent chick provisioning. Nevertheless, we did not find evidence that the level of parental coordination would influence chick growth. Although surprisingly, other authors also did not find a relationship between the degree of nest visit synchrony, reproductive success and/or offspring condition [13, 51]. However, in all these cases, the lowest recorded level of parental coordination might be high enough to secure survival (all chicks survived in the present study) and good body condition of the brood. This may be particularly true in the Dovekie, which has a single-chick brood. In such a case much more time and/or a ‘bigger failure’ may be needed to affect chick growth noticeably. Moreover, it is likely that the effects of various levels of parental provisioning performance on chick growth may be difficult to record when the basic measure of chick body condition is body mass. This parameter has relatively low resolution (i.e. not measured daily) and a high variability. It can also be affected by events that could happen or not happen just before the measurement but were not controlled, e.g. feeding, defecation. We also cannot exclude that the observed effects may be site and/or season dependent. The foraging patterns of the Dovekie depend on oceanographic conditions, with extensions of both LT and ST duration in unfavorable conditions [31]. It is therefore possible that both coordination performance and its effect may be different in another ecological context. An analogous study in different environmental conditions, including various parameters describing chick body condition, would be warranted.

Alternatively, the coordination of foraging trips by breeding pairs may serve primarily to maintain the partners’ body reserves during the energetically demanding chick rearing period, and only secondarily securing regular chick provisioning. Being long-lived birds with long-term pair bonds, both pair members benefit from maintaining themselves in a good body condition [56, 57]. As provisioning is exceptionally costly in the Dovekie [30], both parents doing this in a coordinated manner may economize their effort and spare their body reserves.

Summing up, we provide compelling evidence for the coordinated behavior of breeding partners during the bi-parental period of chick rearing in Dovekie. Dovekie parents provision their chick in a manner that optimizes the time it has to wait for food, supporting the role of cooperation in avian reproductive behavior.

## Supporting information

S1 Table Detailed information on number of valid observations for each Dovekie pair in both seasons. (DOCX)

S1 R-script R-codes (Monte Carlo routines) to analyze coordinated provisioning in the Dovekie. (R)

S1 Data Row data in csv format. (CSV)

S2 Data Legend for S1 Data.csv file. (TXT)
