# Supplementary material for: Seabird parents provision their chick in a coordinated manner
Source: PLoS One. 2018 Jan 10;13(1):e0189969. doi: 10.1371/journal.pone.0189969 (PMC5761830; doi:10.1371/journal.pone.0189969)
Supplement: S1 Table — (DOCX) [file pone.0189969.s001.docx]

**Supplementary materials**

**S1 Table.** Number of valid observations (both members feeding, with well visible mark on breast feathers) for each pair in both seasons

|  | Season | |
| --- | --- | --- |
| Nest | 2009 | 2010 |
| M.F11 | 2 | 3 |
| M.F20 | 2 | 0 |
| M.F22 | 2 | 3 |
| M.F23 | 2 | 1 |
| M.F31 | 0 | 2 |
| M.F33 | 2 | 2 |
| M.F46 | 0 | 1 |
| M.F47 | 0 | 2 |
| M.M20a | 0 | 3 |
| M.M23 | 2 | 3 |
| M.M24 | 2 | 2 |
| M.M25 | 1 | 1 |
| M.M25a | 0 | 3 |
| M.M28 | 0 | 2 |
| M.M29 | 2 | 2 |
| M.M31 | 2 | 2 |
| M.M34 | 2 | 2 |
| M.M35 | 2 | 2 |
| M.M38 | 1 | 0 |
| M.M39 | 1 | 1 |
| M.M40a | 0 | 2 |
| M.M41 | 2 | 3 |
| M.M42 | 2 | 0 |
| M.M43 | 2 | 1 |
| M.M44 | 2 | 3 |
